# The regulatory role of lipophagy in central nervous system diseases

**DOI:** 10.1038/s41420-023-01504-z

**Published:** 2023-07-06

**Authors:** Zhuo-qing Lan, Zi-yi Ge, Shu-kai Lv, Bing Zhao, Cai-xia Li

**Affiliations:** 1 Department of General practice medicine, the Fourth Affiliated Hospital, School of Medicine, Zhejiang University, Yiwu, P.R. China; 2 Department of Anesthesiology, the First Affiliated Hospital, School of Medicine, Zhejiang University, Hangzhou, P.R. China

**Keywords:** Macroautophagy, Autophagy

## Abstract

Lipid droplets (LDs) are the organelles for storing neutral lipids, which are broken down when energy is insufficient. It has been suggested that excessive accumulation of LDs can affect cellular function, which is important to coordinate homeostasis of lipids in vivo. Lysosomes play an important role in the degradation of lipids, and the process of selective autophagy of LDs through lysosomes is known as lipophagy. Dysregulation of lipid metabolism has recently been associated with a variety of central nervous system (CNS) diseases, but the specific regulatory mechanisms of lipophagy in these diseases remain to be elucidated. This review summarizes various forms of lipophagy and discusses the role that lipophagy plays in the development of CNS diseases in order to reveal the related mechanisms and potential therapeutic targets for these diseases.

## Facts

- There are various diseases of the CNS, with complex pathogenesis and difficult treatment.
- Expression of key genes involved in lipophagy regulation in the CNS.
- Improving the prognosis of CNS diseases by regulating lipophagy.

## Open questions

- What is the difference between the regulatory mechanism of lipophagy in the central and peripheral systems?
- What are the specific regulatory mechanisms for lipophagy in the CNS?
- Lipophagy regulatory targets in different neurological diseases.

## Introduction

Autophagy is the process of transferring specific substrates from the cytoplasm to vesicles or lysosomes for degradation and re-circulation. Autophagy also plays a key role in maintaining cellular homeostasis and causing stress and inflammation in tissues [1, 2]. Lipid droplets (LDs) have recently been found to have an intimate association with autophagy. Indeed, LDs can be selectively catabolized through autophagy. In this way, fatty acids (FAs) enhance cellular energy levels [3]. These findings provide a new perspective on the regulation mechanisms of lipid metabolism.

The dysregulation of lipid metabolism has been associated with a variety of central nervous system (CNS) diseases, accompanied by abnormal lipid accumulation with the formation of LDs [4]. Previous studies suggest that the dysfunction of lysosomes and autophagy possibly plays a role in the development of some neurodegenerative diseases [5, 6], suggesting the process of selective phagocytosis of lipids by lysosomes could be involved in the development of CNS diseases.

Lipophagy refers to the process of selective autophagy of LDs through lysosomes. In this cellular process, LDs are degraded through the lysosomal degradative pathway. Although dysregulation of lipid metabolism is associated with a variety of CNS diseases, the specific regulatory mechanisms of lipophagy in these diseases remain unclear. In this review, we summarize various forms of lipophagy and further elucidate the importance of lipophagy in the development of CNS diseases to reveal the related mechanisms and potential therapeutic targets for these diseases.

## Lipid droplets

### The structure of LDs

LDs are spherical organelles with multiple proteins on the surface. LDs consist of a hydrophobic core of neutral lipids (e.g., triacylglycerols (TAG) and cholesteryl esters (CE)), enclosed within a phospholipid monolayer. A representative example of a class I protein is spatacsin, which has been suggested to be involved in the regulation of neurodegeneration [7, 8].

Class II proteins enter the LD surface from the cytoplasm, binding through the amphiphilic helices or other hydrophobic structural domains. One of the most representative members of class II proteins is the perilipin (Plin) family (i.e., Plins1–5). They are involved in the process of LDs movement and signal exchange between organelles, which protect LDs from lipase solubilization. Therefore, Plins can be considered as key factors in the regulation of LDs. Within the Plin family, Plin2, Plin3, and Plin5 are expressed in the human brain, whereas Plin1 and Plin4 are rarely expressed in the CNS [9]. The upregulation of Plin2 expression has been observed in neurodegenerative diseases [9, 10]. In Parkinson’s disease, Plin2 synergizes with α-synuclein (α-Syn), a membrane on the surface of LDs, inhibiting enzymatic lipolysis and promoting the deposition of LDs in the brain [11]. Meanwhile, the expression of Plin3 is enhanced in astrocytes, possibly related to the recruitment of LDs. Furthermore, Plin5 is expressed in other types of cells, such as oligodendrocytes [9].

### The biogenesis of LDs in the CNS

Based on previous findings that the enzymes catalyzing TAG and CE biosynthesis are localized in the Endoplasmic reticulum (ER), the ER has been suggested to be the organelle where LDs are synthesized [12, 13]. FAs are normally transported to the hepatic ER and finally secreted as VLDL or stored as LDs [14, 15]. The process of LD de novo synthesis can be divided into three main steps: (i) nucleation, (ii) growth, and (iii) budding. With the accumulation of TAG and CE, an oil lens structure is formed between the two leaflets of the ER membrane [16, 17]. Afterwards, the small LDs diffuse and fuse with the large LDs. Finally, the LDs sprout out of the ER membrane. The resident protein family BSCL2/seipin was found to be involved in targeted LDs formation [12]. Seipin is a transmembrane protein at the ER-LDs junction, which is also an important structure for LDs formation. Downregulation of seipin significantly increases the level of TAG and aggregation of small LDs [18]. A mutation within seipin might lead to the abnormality of LDs in both shape and number [19, 20]. Importantly, when colocalizing with LC3, seipin has also been associated with autophagy [21]. A mutation within seipin could induce abnormal vesicle generation and eventually affect autophagy [21]. It has been reported that seipin is highly expressed in adult mouse hippocampal CA1 pyramidal cells [22, 23]. The knockdown of seipin in a mouse AD model could inhibit autophagy through the mTOR pathway and increases tau protein aggregation [22]. Therefore, knockdown of neuron-specific seipin in mice induces deterioration in spatial cognition and possibly leads to a variety of neurological disorders [23, 24].

**Fig. 1:**
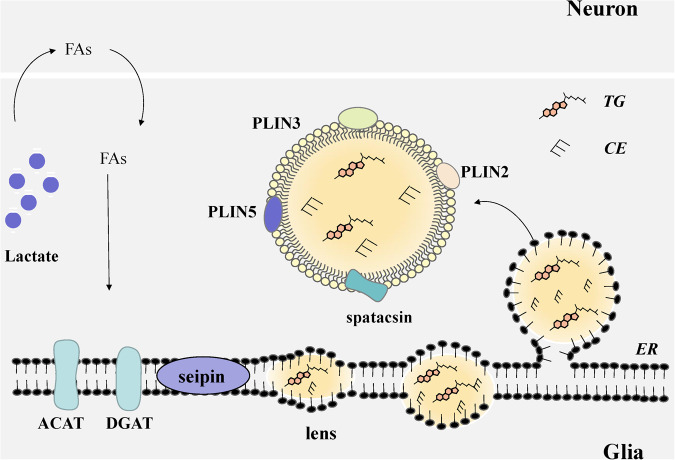
Biogenesis of LDs in general glial cells. During oxidative stress, glial cells take in glucose from the blood and turn glucose into lactate. Lactate is subsequently transported to neurons and converted into FAs. The process of LD de novo synthesis is divided into three main steps: (i) nucleation, (ii) growth, and (iii) budding. Firstly, an oil lens structure forms between two lipid monolayers of the ER, limiting membrane by ER resident proteins such as BSCL2/seipin, which is the key step of nucleation. Afterwards, small-volume LDs diffuse and fuse with the large ones. Finally, LDs sprout out of the ER membrane.

In a healthy state, only a small amount of LDs are visible in the brain, while aging, oxidative stress and various of neurodegenerative diseases could induce a large accumulation of LDs in the brain, especially in glial cells, including astrocytes, microglia, and oligodendrocytes [25]. Conversely, LDs are rarely gathered in neurons, possibly because mitochondria cannot generate enough energy through β-oxidation. Liu et al. [26] proposed the lactate shuttle mechanisms in neuron-astroglial cells. Under pathological conditions, especially in the presence of cellular mitochondrial dysfunction and elevation of reactive oxygen species (ROS), circulating blood glucose is taken up by the glial cells and converted to lactate [26]. Lactate is then transported to neurons via the monocarboxylate transporter protein (MCT) and converted to FAs. Rather than storing these FAs, neurons excrete them into glial cells with APOE-positive lipid particles or fatty acid-associated transporter proteins (FATP) [26–28]. FAs entering glial cells are then directed to ER for esterification. Diacylglycerol acyltransferase (DGAT) 1 and 2 catalyze the reaction of free FAs to TG. Cholesterol acyltransferase 1/sterol O-acyltransferase 1 (ACAT1/SOAT1) is responsible for CE esterification [14, 16]. Nevertheless, APOEε4 (a specific genetic variant) disrupts FA metabolism coupled between neurons and astrocytes [26]. For PD patients, LDs appear to relocate between the neurons and glial cells [29]. This pattern indicates the significance of maintaining lipid homeostasis. The structure of LDs and biological processes in the CNS are presented in Fig. 1.

### Function of LDs

LDs have traditionally been considered as sites for resident neutral lipids. During starvation or cell growth, FAs in LDs can be catabolized by lipolytic or lipophagic pathways to allow for cell membrane expansion or biosynthesis of other lipid species, subsequently, participate in β-oxidation to provide metabolic energy [30]. LDs have been re-recognized as being involved in lipid metabolism, protein storage, and signaling regulation [31]. Interestingly, the number of LDs increases during prolonged nutrient deprivation or under oxidative stress, possibly due to the prevention of lipotoxicity by isolating free FAs, such as ceramide, acylcarnitine, and diacylglycerol [32]. LDs are then degraded by organelle autophagy, which might provide a platform for lipid buffering [32]. It is worth noting that LDs have recently been suggested to be the first line of defense against bacterial invasion [33]. When dangerous bacteria enter cells, immune proteins on LDs (such as viperin, IGTP, IIGP1, TGTP1, and CAMP, etc.) are activated immediately to kill pathogens in a synergistic manner. LDs also connect with the membrane contact sites of other organelles. This is of great importance for maintaining lipid metabolic homeostasis and achieving energy homeostasis. Apart from interacting with the ER and promoting production of LDs [34]. LDs also interact with mitochondria in a Plin5-dependent manner. As such, the physical distance reduced between LDs and the mitochondria which significantly improves the efficiency of FAs transferred to mitochondria for β oxidation [35].

### Catabolism of LDs: lipolysis

Lipolysis refers to the hydrolysis of TG into free FAs and glycerol in LDs for providing energy or preventing lipotoxicity. Lipolysis is usually divided into two parts: neutral lipolysis outside the lysosome and acidic lipolysis inside the lysosome, depending on the pH value and the organelle where lipolysis takes place. At the neutral pH, neutral lipase directly acts on LDs located in the cytoplasm and decomposes TAG and CE stored in LDs. Three major neutral lipases include (i) ATGL/PNPLA2, (ii) HSL, and (iii) MGL. ATGL/PNPLA2 is the rate-limiting enzyme that catalyzes the first step of TG hydrolysis. In the presence of peroxisome proliferator-activated receptor (PPAR) agonists (e.g., glucocorticoids or starvation), the expression of ATGL/PNPLA2 is upregulated. It has been reported that the rapamycin-sensitive complex (mTORC1) dependent signaling pathway reduces ATGL mRNA levels, whereas sirt1-mediated deacetylation elevates ATGL expression by activating FOXO1 and consequently promotes lipolysis [36, 37]. HSL mainly regulates the process of DAG degradation, as well as the degradation of other lipids, such as MAG, cholesteryl esters, and retinyl esters, etc. MGL catalyzes the hydrolysis of MAG to glycerol [36]. Besides, there is a specific triglyceride enzyme named DDHD2 that is actively expressed in the brain and less frequently in peripheral tissues. Knockdown of DDHD2 in mice could lead to the accumulation of a large amount of TG in the brain and the formation of LDs in neurons.

Moreover, lipolysis that occurs in lysosomes is mediated by lipases such as LIPA/LAL (lipase A and lysosomal acid). The importance of LAL in lipid metabolism has been demonstrated in both mice and humans. Deletion and mutation of the LIPA gene could lead to rare genetic diseases such as Wolman disease. As the function of LAL is completely lost, Wolman disease manifests with accumulation of a large amount of CE and TG in cells, which could lead to subsequent tissue damage [38]. Similarly, the function of LAL only preserves 5–10% in CE storage diseases. This could lead to a number of clinical manifestations, including hyperlipidemia, hepatosplenomegaly, premature atherosclerosis, and coronary artery diseases [38]. In contrast with lipoprotein-associated TAG that is degraded via early and late endosomal transport to lysosomes, neutral esters are usually degraded in lysosomes in a lipophagic manner.

## Lipophagy

**Fig. 2:**
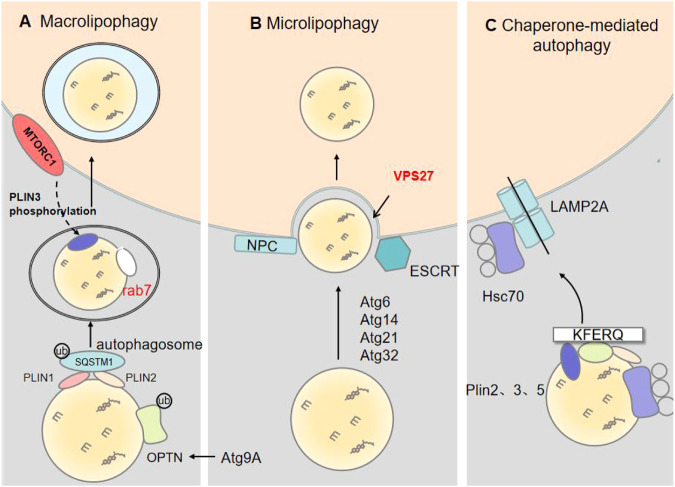
The specific regulatory mechanisms of lipophagy. **A** Macrolipophagy involves the sequestration of LDs by autophagosomes and subsequently delivers them to lysosomes/vacuoles for turnover. On the one hand, Plin1 and Plin2 bind with macroautophagy cargo receptor SQSTM1/p62, which triggers Ub-dependent macrolipophagy. On the other hand, Atg9A regulates OPTN (an autophagy-selective receptor) and leads to Ub-dependent macroautophagic degradation of LDs. Furthermore, Rab7 interacts with mTOR through its N-terminal hot repeat structural domain, and mediates lipophagy by regulating the mTOR signaling pathway. mTORC1 regulates lipophagy through a Plin3 phosphorylation dependent mechanism, and possibly inhibits lipophagy through the ULK1 pathway. **B** Microlipophagy has been better characterized in yeast. Microlipophagy relies on the formation of sterol-enriched vacuolar microdomain. This microdomain takes up LDs in a manner dependent on proteins such as Atg6, Atg14, Atg21, and Atg32. In addition, microlipophagy is regulated by ESCRT, no matter whether it depends on Atg or not. ESCRT drives the invagination in the vacuolar membrane, and interacts with clathrin proteins through one of its components (Vps27) to mediate phagocytosis. **C** In chaperone-mediated autophagy, Hsc70 recognizes proteins possessing a KFERQ motif, including Plin2, Plin3, and Plin5. This cargo-chaperone complex subsequently binds with LAMP2A and causes LD degradation.

Lipophagy is a specific form of autophagy in which CE and TAG in LDs are turned to generate free FAs by activating autophagy-associated molecules. Recent findings about the colocalization of autophagy and LD markers further confirm the presence of lipophagy in lysosomes [4]. Lipophagy consists of three main processes: (i) microtubule-associated protein 1 light chain 3 (LC3) recognizes autophagic receptors on LDs, (ii) LC3 and Atg1/ULK1 complex (including Atg1, Atg13, Atg17, etc.) facilitate the formation of autophagosomes, and (iii) Lipid autophagic vesicles eventually fuse with multivesicular bodies with intraluminal vesicles or lysosomes to form autophagic lysosomes that are finally hydrolyzed in lysosomes by acid lipase [39]. Conversely, the occurrence of lipophagy can be blocked by the application of lysosomal inhibitors [40]. Lipophagy is mainly degraded through macroautophagy, microautophagy, and chaperonin-mediated autophagy (CMA). The specific regulatory mechanisms of lipophagy in the CNS are summarized in Fig. 2.

### Macrolipophagy

Macrolipophagy is a typical type of macroautophagy that refers to the formation of autophagosomes with double membrane vesicles to encapsulate intracellular materials and eventually fuse with lysosomes. Singh et al. [3] reported that LC3 is recruited into LDs to form a restricted membrane in an autophagy-associated protein 7 (Atg7)-dependent manner and, subsequently, generates autophagosome in a phagocytosed bilayer. Finally, a matured autophagosome fuses with lysosomes and exposes the engulfed cytoplasmic material to acidic lysosomal hydrolases for degradation. Therefore, LDs are considered to be degraded via a macroautophagy-dependent pathway. Interestingly, the autophagosome can only encapsulate small LDs but not large LDs [41]. It was reported that inhibiting autophagy leads to the accumulation of small LDs inside the lysosome, and lipolysis can rapidly shrink large LDs to a diameter that is more suitable for being engulfed by lipophagic vesicles [41].

SQSTM1/p62 (isolated vesicle 1) is a selective autophagy receptor, where the ubiquitin-associated (UBA) structural domain and the LC3 interaction region (LIR) mediate its function [40]. It was reported that the association of both SQSTM1 and LC3 with LDs is significantly reduced when Plin1 is knocked down [42], suggesting that SQSTM1 and LC3 colocalize with the LDs surface protein Plin1 under ethanol-induced stimulation condition. However, classical selective autophagy is ubiquitin-dependent, since ubiquitin signaling on LDs and co-localization of SQSTM1. As Plin1 has previously been reported to be regulated by polyubiquitination [43], ubiquitinated Plin1 could possibly be a target for SQSTM1 recognition and promotes macroautophagy. There was another study reporting that the addition of rapamycin-induced myoblast lipophagy in a skeletal muscle cell line (L6 myoblasts) triggers the binding of SQSTM1 to Plin2 on the surface of LDs. Results of these studies indicate that SQSTM1 engulfs LDs into autophagosomes via the Plin2 pathway. Co-accumulation of SQSTM1 and LDs can be observed when lipophagy is inhibited with bafilomycin A1 or chloroquine by preventing the fusion of lysosomes with autophagosomes [43, 44]. This suggests that SQSTM1 is degraded in lysosomes together with LDs. Huntingtin protein (Htt) is a scaffolding protein that is involved in selective autophagy. Htt has been suggested to interact with SQSTM1 and promote its association with LC3. Mutation in Htt could cause the accumulation of autophagic vesicles and cytoplasmic LDs [44].

Another common autophagy-selective receptor is optineurin (OPTN). OPTN was reported to be co-localized with ubiquitin and LC3 on LDs, and knockdown of OPTN in foam cells leads to a significant reduction of lipophagy-driven CE efflux [45]. Conversely, OPTN is dramatically increased after the application of chloroquine treatment on the surface of LDs [45]. Furthermore, OPTN expression has been reported to be upregulated in patients with hepatocellular carcinoma and nonalcoholic fatty liver disease (NAFLD). Adipogenic efficiency was significantly reduced after the knockdown of OPTN in HepG2 cells by siRNA, suggesting that OPTN can delay adipogenesis in a fatty liver by lipophagy [46]. It was reported that knockdown of the autophagy related 9 A (Atg9A) gene in human cells results in an increase in the number and size of LDs [47]. After knockdown of the Atg9A gene, the zinc finger structure in OPTN presents the autophagic function interacting with Atg9A, suggesting that OPTN could be regulated through Atg9A [48]. Atg9A is the only multi-transmembrane protein among the Atg proteins that is essential for autophagic vesicle formation. As OPTN has been suggested to play a role in the control of lysosomal mass, this further ensures autophagic flux. Studies in cholesterol-rich SH-SY5Y cells and cultured primary neurons revealed that high intracellular cholesterol levels could induce defective recruitment of OPTN and impaired lysosome-mediated clearance [49]. Although the specific regulatory mechanisms of lipophagy have not been fully elucidated, the small Rab proteins belonging to the Ras superfamily are thought to be directly involved in regulating lipophagy. Rab7 is the first member of the Rab family that was found to be involved in lipophagy. Rab7 has also been associated with LDs, autophagic membranes, and lysosomes under starvation conditions [50]. Silenced Rab7 expression or decreased activity results in the accumulation of LDs in cultured hepatocytes [50]. When stimulated by β-adrenergic receptor activation-mediated lipolysis, the recruitment of Rab7 to LDs and autophagosomal membranes was observed [51]. However, the occurrence of macrolipophagy was inhibited by Rab7 depletion or inactivation [51]. Rab7 also plays a role in membrane transportation, which is essential in regulating the maturation of early endosomes to late endosomes, the translocation and fusion of late endosomes to lysosomes, as well as the progression of lysosomogenesis. Rab7-labeled LDs can be observed in both Toxoplasma gondii and Mycobacterium tuberculosis-induced infections. These observations suggest that Rab7-mediated lipophagy is present in different infections [52, 53]. Rab7 was found to interact with mTOR through its N-terminal hot repeat structural domain, and deletion of Rab7 GTPase in myeloid cells leads to a remarkable decrease of LAMP1 expression and concomitant downregulation of the mTOR downstream signaling pathway in MDSC-like HD1B cells [54]. This possibly indicates that Rab7 not only controls lysosomal genesis but also mediates lipophagy by regulating the mTOR signaling pathway [54]. Besides, it has been reported that Rab7 deletion significantly impairs the recruitment of Rab10 to the autophagic membrane around LDs, while Rab10 deletion does not affect the localization of Rab7 [55]. This suggests that Rab10 localizes to LDs and autophagosomal membranes in a Rab7-dependent manner, facilitating the extension of autophagic vesicle membranes around LDs for phagocytosis by stimulating the binding to the bridging protein EHBP1 (EH structural domain binding protein 1) and the membrane deforming adenosine triphosphatase EHD2 (EH structural domain containing 2). Similar to Rab7, deletion of Rab10 leads to the accumulation of LDs in starved hepatocytes [55]. Furthermore, Rab18 was also suggested to be associated with lysosomes and involved in lysosomal transport and autophagy. Knockdown of Rab18 by shRNA leads to a decrease in autophagic activity, while the overexpression of Rab18 enhances autophagy and in turn affects lipid degradation. Rab18 was suggested to colocalize and co-separate with Rab7 on lysosomes. The expression of Rab7 was elevated in Rab18 knockdown neurons, suggesting a possible compensatory effect. Therefore, Rab7 and Rab18 might play a synergistic role in lysosomal functioning and autophagy [56]. During LDs accumulation, Rab18 translocates from the ER to the LDs and, in turn, degrades Plin2, thereby inducing lipophagy [57]. However, lipolysis is diminished after the application of autophagy inhibitors [57].

MTORC1 on the surface of the lysosome has a vital impact on cellular lipid homeostasis. MTORC1 consists of mTOR, raptor, mLST8, and PRAS40 subunits, and the raptor subunit is one of the most unique components. The raptor subunit is a scaffolding protein for the entire complex and plays a crucial role in recruitment as well as other activities involving the complex components. Raptor subunit can be acetylated as a target of EP300, involved in the regulation of autophagy. It has been indicated that mTORC1 promotes lysosome synthesis through TFEB (transcription factor E3) and regulates autophagy through activation of ULK [58]. With the application of rapamycin (i.e., an inhibitor of mTORC1), hepatocytes reduce the accumulation of LDs in an autophagy-dependent manner [59]. Knockdown of the raptor subunit on mTORC1 promotes the degradation of LDs in terminally differentiated cells; this could be rescued by inhibiting lysosomal function or knocking down Atg7 [60]. Furthermore, the raptor subunit was found to interact with Rag proteins and translocate mTORC1 to the lysosomal surface in a Rag protein-dependent manner. LDs are accumulated when the activities of RagA and RagB in the lysosome are blocked. Hence, LDs could be degraded during lipophagy, and mTORC1 is also involved in this process. It was reported that mTORC1 regulates lipophagy through a Plin3 phosphorylation dependent mechanism, and the application of rapamycin or silencing mTOR could enhance the accumulation of Plin3 on the LDs and inhibit lipophagy [61]. As knockdown of Raptor increases ULK1 phosphorylation in adipocytes, mTORC1 possibly inhibits lipophagy through the ULK1 pathway [60]. Although mTORC1 relies on Plin3 to regulate lipophagy, Plin3 does not contain a recognized LC3-interacting region (LIR) to mediate its direct binding to LC3, suggesting Plin3 might mediate the onset of lipophagy through other pathways.

In Plin3-silenced NIH-3T3 cells, the autophagic flux of LDs-associated LC3-II and LAMP1 is disrupted and the accumulation of autophagic proteins on LDs is strongly inhibited, whereas the application of oleic acid (OA) and lysosomal inhibitors increases the co-localization of LC3 and LAMP1 with LDs. These results further demonstrate that Plin3 is required for lipophagy [61]. Phospholipase D1 (PLD1), a phosphatidic acid producing enzyme, is recruited in lysosomes and consequently involved in LD accumulation during nutrient starvation. RalA acts downstream of autophagy by recruiting PLD1. RalA is subsequently involved in the recruitment of Plin3 and induces aggregation of LDs, and the inhibition of RalA prevents the formation of LDs. These results indicate the important role of Plin3 in lipophagy [62].

### Chaperone-mediated autophagy

The Plins located on LDs physically prevent lipophagy and lipolysis of TG and CE. Both Plin2 and Plin3 carry KFERQ-like peptide motifs recognized by heat shock homologs (HSPA8/Hsc70), and then bound and translocate to lysosomes. This process is known as chaperone-mediated autophagy, abbreviated as CMA [63] During starvation, CMA is enhanced and exhibits enrichment with an increase in both cytoplasmic ATGL levels and macroautophagy-associated proteins [64], possibly indicating that CMA-mediated degradation of LDs requires the coordination of lipolysis and macroautophagy.

Recent studies reported that Sirt3 promotes the CMA process and decreases the stability of LDs [65]. The expression of Sirt3 increases LAMP-2A-tagged lysosomal contents while decreasing Plin2 levels. When Hsc70 is downregulated, the levels of both LAMP-2A and Plin2 proteins are significantly increased. These results suggest that Sirt3 overexpression possibly promotes the CMA process and facilitates the degradation of Plin2 in LDs. Sirt3 was also found to strengthen macroautophagy via the AMPK-ULK1 pathway, and AMPK knockdown similarly reverses the decline of Plin2. Therefore, Sirt3 possibly promotes macroautophagy by activating AMPK in synergy with CMA and eventually contributes to the degradation of LDs [65].

Plin5 has been reported to be another substrate for CMA degradation in the mouse liver. Once lysosomal activity and CMA-mediated Plin5 degradation are inhibited in mouse liver or HepG2 cells, the function of LDs could be impaired [66]. Taken together, these studies suggest that CMA-mediated Plin protein degradation can be considered to be a result of the involvement of lipolysis and macrolipophagy, emphasizing the importance of the interactions between lipophagy, lipolysis, and lysosomes.

### Microlipophagy

The direct phagocytosis of LDs by endonucleosomal processes in yeast is known as microlipophagy. During nutrient deprivation, a sterol-enriched vacuolar microdomain is formed on the vesicle membrane of yeast. This microdomain in turn directly takes up LDs in a manner dependent on proteins such as Atg14p, Atg6p, Atg21, and Atg32 [67]. This procedure does not rely on the typical macroautophagic approach, because there is no core component such as Atg7 involved in this process [68, 69]. Similarly, microlipophagy is confirmed by the observation that liphagy cannot be induced by treatment with rapamycin in yeast [70]. Nevertheless, it has been reported that the occurrence of Atg proteins involved in lipophagy changes with environment. For instance, the vesicular membrane protein Atg22 (responsible for the efflux of amino acids from vesicles) is essential for stationary phase lipophagy but is not necessary for lipophagy induced by acute carbon starvation [68, 70, 71].

Atg proteins are not necessary for lipophagy induced by phospholipid imbalance [68]. Microlipophagy functions independently of Atg proteins. The endosomal sorting complex required for transport (ESCRT) consists of membrane-associated proteins involved in membrane break events that participate in the repairing processes when lysosomal stress is impaired. ESCRT has been considered to be associated with microlipophagy [72]. The microlipophagic pathway, no matter whether Atg proteins are dependent or not, is regulated by ESCRT [73]. ESCRT is localized to vesicles, driving the invagination of the vacuolar membrane. ESCRT also interacts with clathrin proteins through one of its components, Vps27, to mediate phagocytosis. The microlipophagic process would be damaged in the absence of Vps27 [74]. Furthermore, several studies have shown that lysosomal cholesterol transporter protein Niemann-Pick C 1/2 (Npc1 and Npc2) is required for macrolipophagy [75]. Especially, Npc2 is thought to play an important role in raft-like microdomain formation during progressive nutrient depletion or nitrogen starvation. Npc2 is likely to promote microlipophagy by increasing sterols in the vesicle limiting membrane.

Besides yeast, the fungus *S. aeruginosa* can also modulate the degradation of attached intracellular LDs by microautophagy, which rapidly forms intracellular expansion pressure to penetrate the insect host body wall [76]. After the relevant autophagy genes of the entomopathogenic fungus *S. aeruginosa* mutate, LDs in the mutant strain can still enter the vesicles without being wrapped by autophagosomes, indicating accumulation rather than degradation [76]. Emerging studies have shown a new method of autophagic LDs degradation in hepatocytes, named “direct lysosome-based autophagy” [69]. In this process, stable contacts between LDs and lysosomes in hepatocytes can be transferred directly from LDs to lysosomes under nutrient-limited conditions in the absence of autophagic intermediates. This phenomenon is preserved even when the core macroautophagy (Atg5) or CMA (LAMP2A) components are knocked down by siRNA [69]. Taken together, these findings suggest that microautophagy pathway-mediated LDs degradation is widely present in a variety of cells under different conditions.

## The role of lipophagy in CNS diseases

Lipophagy has been suggested to be involved in the development of CNS diseases such as ischemic encephalopathy as well as cerebrovascular and neurodegenerative diseases. The related mechanisms remain unclear. The relevant aspects of these diseases in vitro and in vivo are summarized in Table 1. Challenges and advances of lipophagy in CNS diseases are discussed in the following section.

**Table 1:** The relevant aspects of lipophagy in CNS diseases.

Animal models	vitro or vivo	Type of modeling	Cells type	Author and year of publication	Summary of findings
Mouse	Vitro	Tuberous sclerosis	Neural stem cells	[79]	Lipophagy functions in coupling energy availability and exacerbates TSC pathogenesis.
	Both	Huntington’s disease	Embryonic fibroblasts	[106]	Impaired microlipophagy leads to the aggregation of LDs.
	Vitro	Amyotrophic lateral sclerosis	Embryonic fibroblasts and neurons	[119]	The loss of spatacsin leads to the accumulation of lipids in lysosomes by perturbing their clearance from the organelles.
	Both	Amyotrophic lateral sclerosis	Embryonic fibroblasts and cortical neurons	[122]	The loss of spatacsin disrupts calcium homeostasis and prevents the clearance of cholesterol from the lysosome.
	Both	Parkinson’s disease	SH-SY5Y cell	[127]	Linoleic acid stimulates the biogenesis of LDs and improves lipophagy flux in the in vitro PD model.
	Vivo	Parkinson’s disease	BE(2)-M17 cell	[128]	Mutations in GBA generate the accumulation of sphingolipids and cholesterol in lysosomes, subsequently leading to autophagy impairment.
	Both	Alzheimer’s disease	Microglia	[129]	The microglial plays a critical role in regulating lipid homeostasis and tau pathology via autophagy.
Rat		Ischemic-reperfusion	Brain endothelial cells	[83]	Lipophagy leads to CE hydrolysis and increased free CE secretion, contributing to several transient ischemia.
Drosophila	Vivo	Huntington’s disease	Fibroblasts	[44]	Lipophagy is required in HD.

### Tuberous sclerosis

Tuberous sclerosis (TSC) affects multiple organ systems and promotes tumor formation in many different organs. TSC particularly influences the CNS and possibly causes seizures, autism spectrum disorders, and cognitive dysfunction [77]. TSC is considered to be caused by a functional mutational deletion of the TSC1/2 gene. In a healthy state, TSC1 and TSC2 form the TSC protein complex and induce phosphorylation of ULK1 at the S757 site and inhibit the mTORC1 signaling pathway. Thereby, TSC plays a critical role in regulating autophagy and lipid homeostasis [78]. Defective TSC in neurons increases autolysosome accumulation and autophagic flux. Moreover, the knockdown of TSC in neurons counteract mTORC1 inhibition through AMPK-dependent ULK1 activation, promoting autolysosome accumulation and elevating autophagic activity. This could be a self-protective procedure of the organism to provide the body with energy for the purpose of resisting prolonged cellular stress through the activation of autophagy. Nevertheless, with the knockdown of TSC in neural stem cells, autophagy could both maintain intracellular energy requirements and the hyperactivation of mTORC1. mTORC1 hyperactivation in turn exacerbates the abnormal differentiation of neural stem cells. Fip200 is required for neoplastic phenotypes driven by mTORC1 hyperactivation. Blockage of Fip200 activity would cause inhibition of lipophagy. consequently, the function of TSC-deficient neural stem cells is restored and tumorigenesis is suppressed [79]. This indicates that modulation of lipophagy or inhibition of other autophagic pathways could be potential therapeutic strategies for treating TSC neuronal pathology.

### Cerebral ischemia

Stroke induced by cerebral ischemia is the most common cerebrovascular disease, which is a common cause of permanent disability [80]. In cerebral ischemia, LDs have been demonstrated to be accumulated in the brain, which then plays a proinflammatory and pro-death role in an ischemic brain [81]. Autophagy widely exists in the human brain, which helps the body to adapt to environmental stress and to remove abnormal substances. In mouse models, ischemia was found to promote autophagy and increase the expression of associated Atg protein in PC12 cells [82]. Furthermore, LC3-II was observed to co-localize with LDs in cellular models of ischemia and hypoxia, suggesting that the involvement of lipophagy in the adaptive processes of the organism attenuates brain damage [83]. In rat brain endothelial cells, lipophagy promotes CE hydrolysis and frees CE secretion. This could contribute to the alteration and/or adaptation of the blood brain barrier in response to the cumulative effects of several transient ischemia [83]. Nevertheless, the abnormal lipophagy could also exacerbate disease progression. Emerging studies have reported that ferroptosis induces and aggravates brain damage following cerebral ischemia [84]. As lipid peroxidation is closely related to ferroptosis, these findings suggest that lipophagy provides a substrate for lipid peroxidation during ferroptosis, which worsens the disease by inducing lipid release and subsequent lipid peroxidation [85]. Furthermore, emerging evidence has suggested that PLIN2,RAB7A and ATG5 mediating lipophagy regulation are the key points in modulating cellular sensitivity to ferroptosis [86]. Although emerging evidence reinforces the idea that lipophagy affects the ability of inducing ferroptosis in other systems, the role of lipophagy in the nervous system has not been revealed yet, which could be a promising field for the treatment of neurological diseases.

### Aging

Aging is a biological process characterized by time-dependent cellular and functional decline. Aging in the brain is accompanied by a decline in the function of the ubiquitin-proteasome system and the autophagy-lysosome pathway. Beclin 1, LC3-II, and LC3-II/LC3-I ratios and Hsc70 concentration in cerebrospinal fluid often decline with age [87]. Consistent with this finding, macroautophagy in the CNS was also found to exhibit an age-related decline [88]. Furthermore, abnormal lipid deposition can be observed in an aging brain [89]. For instance, the accumulation of LDs and colocalization with Beclin-1 or LC3 has been reported in aging brain cells including the microglia, astrocytes, neurons, and ventricular tract cells [89]. The lipid droplet-rich microglia, known as the LDs aggregating microglia (LDAM), has also been found in aging brains. LDAM exhibit an enhanced inflammatory response and reduced phagocytosis, which could increase expression of the LDs surface protein Plin2 [10]. These results suggest that both macrolipophagy and CMA-mediated lipophagy might be impaired with aging. Meanwhile, both autophagy and LIPL-4-dependent lipolysis were found to be highly expressed in germline-less *C. elegans* [90, 91]. They work interdependently to prolong the life span. As LIPL-4 is thought to be a homolog of mammalian LIPA, aging might have a similar influence on acidic lipolysis in the lysosome.

### Neurodegenerative diseases

#### Parkinson’s disease

Parkinson’s disease (PD) is a common, complex, and progressive neurodegenerative disease. The main neuropathological features of PD are considered to be the degeneration of dopaminergic neurons in the substantia nigra (SN) and the abnormal aggregation of misfolded α-synuclein (α-syn) within Lewy body [92]. Lipid metabolism has been suggested to be disturbed in PD patients taking untargeted metabolomics and proteomics [93]. Therefore, LDs are increasingly recognized as critical organelles with important effects on neuronal activity in PD. The aggregation of LDs within dopaminergic neurons is also observed in PD animal models [94]. α-synuclein (α-syn) is a lipid-binding protein that interacts with phospholipids and FAs. α-syn has been suggested to play a critical role in the neurodegenerative process of PD by impairing multiple subcellular functions [95, 96]. The expression of α-syn leads to an increase in the levels of unsaturated FAs (UFAs), especially oleic acid (OA). The increased UFAs could in turn worsen the toxicity of α-syn. Inhibition of OA-producing enzyme stearoyl-CoA desaturase (SCD) has been suggested to promote the formation of αS polymer and inhibit its toxicity, and SCD knockout models in roundworms was observed to prevent dopaminergic neuron degeneration [97]. In addition, α-syn was found to bind to LDs, and the overexpression of α-syn can lead to the accumulation of LDs [98]. The above findings indicate a close relationship between α-syn and lipid metabolism imbalance, which is supported by Fanning et al. (2020) who stated that α-synuclein toxicity and cell trafficking defects have been associated with aberrations in LDs content and distribution. This idea has also been supported by previous studies revealing that α-syn nucleoprotein toxicity and cellular transport defects are associated with the abnormal content and distribution of LDs. Meanwhile, α-syn can be degraded through CMA as well as macroautophagic pathways. As a result, inhibiting CMA or macroautophagy could lead to α-syn aggregation in neurons [99, 100]. The expression of CMA core proteins (i.e., lysosomal-associated membrane protein 2 [LAMP2A] and HSPA8) was also reported to be significantly decreased in PD patients [101].

GPNMB is a phagocytic protein that is required for recruiting LC3 to phagocytic vesicles. GPNMB usually colocalizes with phagocytic vesicles and promotes lysosomes to fuse with them. GPNMB expression significantly increases the rate of acidification of phagocytic vesicles caused by lysosomal fusion [102]. A substantial genome-wide association study (GWAS) reported that the GPNMB gene is greatly associated with idiopathic PD. Inhibiting GBA1 in mice alters the glycolipid levels and causes an elevation of GPNMB. The accumulation of glycolipid severely alters membrane fluidity and lipid raft structure and then leads to alpha-synucleinopathy [103]. Furthermore, phospholipid content in lipid has been suggested to directly influence the aggregation capacity of α-syn [103, 104]. Taken together, lipid metabolism disorders and lysosomal dysfunction are critical to induce GPNMB alterations.

#### Huntington’s disease

Huntington’s disease (HD) is a neurodegenerative disease characterized by motor and cognitive deficits resulting from mutations in Htt. This mutation leads to the aggregation of Htt, and, subsequently, neuronal death [105]. A distinct increase in LDs can be observed in mice neurons that express Htt [106]. This phenomenon is possibly caused by the impairment of macroautophagy, because the increase in LDs was reported to slow down after the blockage of lysosomal degradation [106]. It was reported that Htt interacts with the autophagic cargo receptor p62 and facilitates the association between LC3 and lys-63-linked ubiquitin-modified substrates in mammalian cells [107]. Htt is therefore considered to regulate lipophagy by negatively regulating mTOR and releasing ULK1. Furthermore, the activity of FOXO (i.e., a transcription factor that regulates macrolipophagy) is also altered in HD [107]. Therefore, lipophagy plays a critical role throughout the occurrence and development of HD.

#### Alzheimer’s disease

Alzheimer’s disease (AD) is the most common cause of dementia. The main features of AD include the accumulation of extracellular amyloid β (Aβ) and intracellular hyperphosphorylation of microtubule isolated protein tau (MAPT) [108]. LDs have been reported to accumulate in ventricular membrane cells of transgenic AD mice and postmortem AD patients. The accumulation of LDs exacerbates the development of pathology and lysosomal dysfunction, thus affecting autophagy [109, 110]. Under oxidative stress, neurons can export lipid precursors to astrocytes via APOE and then degrade those lipid precursors. Increasing evidence has shown that functions differ among different ApoE subtypes. In particular, APOE-4 is a major genetic risk factor for AD. The inability of APOE-4 to transport lipids could lead to LD formation in neurons, and the accumulation of LDs subsequently promotes the development of AD [26]. Furthermore, other risk genes of AD are consistent with lipid transportation mechanisms; they include the lipid transporters ABCA1 and ABCA7 in neurons, the APOE receptor LRP1 in glial cells, and some endocytosis genes in glial cells, such as PICALM, CD2AP, and AP2A [111]. Those genes are possibly responsible for the deposition of LDs in AD patients. Notably, PICALM can regulate autophagy and affect the clearance of tau protein, and the altered PICALM expression could exacerbate tau-mediated toxicity in both zebrafish and transgenic mice models [112, 113]. Meanwhile, PICALM is involved in the nucleation and transport of lipids, as mediated by lipoprotein particles containing APOE and clusterin [114]. LDs have been suggested to precede the formation of amyloid plaques and neurofibrillary tangles [109]. Impaired autophagy in mice neurons was observed to advance the development of Aβ plaques, which could result from insufficient lysosomal acidification. Taken together, these results indicate that attenuated lipophagy possibly induces the formation of LDs and exacerbates AD in an early stage.

#### Amyotrophic Lateral Sclerosis

Amyotrophic lateral sclerosis (ALS) is a neurodegenerative disorder characterized by progressive degeneration of motor neurons. LDs have been suggested to play a critical role in the development of ALS pathophysiology. Genes that are responsible for ALS usually play important roles in the biology of LDs, and the disruption of lipid metabolism and energy homeostasis are prevalent in ALS [115, 116].

The hexanucleotide repeat expansion in C9orf72 is the most common cause of ALS [117]. C9orf72 is required for lysosomal targeting and degradation of coactivator-associated arginine methyltransferase (CARM1), which is an important epigenetic regulator of macroautophagy and lipid metabolism. Therefore, C9orf72 is considered to be a critical regulator in the autophagy-lysosome pathway for negative feedback control during nutrient stress response [118]. Deficiency in C9orf72 could lead to an increase in LDs and lipophagy that is consistent with increased autophagic flux [118]. Recent studies suggested that SPG11 could contribute to an early onset form of ALS [119]. SPG11 knockout mice have a large accumulation of lipids and p62 in their lysosomes and present similar motor and cognitive symptoms as those observed in ALS patients [119]. Spatacsin (a protein encoded by SPG11) is possibly responsible for this phenomenon [120]. Spatacsin has been suggested to be involved in autolysosome reformation and autophagic clearance [121]. Analysis of SPG11 knockout mice suggested that the loss of spatacsin function could lead to progressive accumulation of lipids in lysosomes, in both neuronal and non-neuronal cells [119]. Loss of spatacsin inhibits the formation of tubules within lysosomes and prevents the clearance of cholesterol and, subsequently, promotes the nuclear translocation of the master regulator of lysosomal TFEB [122]. The activation of TFEB can be considered to be a compensatory mechanism that maintains lipid balance by enhancing lipophagy. Therefore, lipophagy plays a complex and critical role in ALS.

### Brain tumors

Due to the enhanced glycolysis and lipogenesis under normoxic conditions, LDs have been reported to accumulate in the brain tumor cells. Previous studies suggested that LDs participate in the formation and development of tumors, and play an important role in signal transduction between tumor cells [123]. Therefore, LDs have been emerging as a promising target for the treatment of brain tumors.

#### Glioblastoma

Glioblastoma (GBM) is the most common and malignant brain tumor in adults. Despite there are many biological and pharmacological approaches for GBM treatment, the therapy resistance feature of GBM inevitably leads to refractory tumor recurrence and treatment failure [124]. Recent studies have reported that LDs can sequester lipophilic drugs and prevent them from reaching the targets, eventually reducing the efficacy of drugs. As a result, inhibition of LDs accumulation could improve the effectiveness of treatments in GBM [125]. Recently, one study about GBM cells with mTORC1 hyper-activation provides insights into the significance of lipophagy in GBM cells metabolism and tumour progression [126]. The above study suggested that GBM cells maintain energy supply through lipophagy and induce hyper-activation of MTORC1, finally leading to a poor prognosis for brain tumors [126]. Therefore, targeting on lipophagy inhibition might be an efficacious treatment for malignant GBM. Interestingly, when using pharmacological approaches to induce the activation of autophagy and the malfunction of lysosome, massive lipids accumulate in the lysosome and finally cause GBM cells death [126].

## Outlook

LDs are an important organelle in the nervous system. Understanding the regulatory mechanisms of LD has profound implications for the development and progression of CNS diseases. Lipophagy selectively degrades LD within the cell and maintains intracellular lipid metabolic homeostasis. Insufficient or excessive intracellular lipophagy not only leads to lipid metabolism disorders in the human body but also possibly causes development and progression of CNS diseases via a variety of mechanisms. The molecular mechanisms are diverse, but all of them are achieved through lipophagy to degrade TAG and CE in LD. Therefore, investigating the role of lipophagy in CNS diseases and exploring the specific regulatory mechanisms provides new potential targets for prevention and treatment of neurological diseases.

## Data Availability

We confrm that the text and figures in this review are original. There is no original research data disclosed or included in this review.
